# The More the Merrier: Multiple Genomes in a Parasitic Prokaryote

**DOI:** 10.1371/journal.pbio.0040212

**Published:** 2006-05-30

**Authors:** Liza Gross

The notion that bacteria and other single-celled “prokaryotes” carry only one genome copy per cell is as ingrained as the notion that they lack a distinct nucleus. (Organisms with one genome copy are “haploid”; organisms with two, including humans, are “diploid”; and organisms with more than two, such as plants, are “polyploid.”) This assumption is based on decades of research on *Escherichia coli* and a select group of other bacterial species.

But researchers have been finding indications that some bacteria, under certain conditions, have more genetic material in a single cell than would be expected for a single genome. And now, using a combination of cell sorting, fluorescent microscopy, microarray, and quantitative PCR techniques, Deborah Tobiason and Hank Seifert present strong evidence that the bacterium that causes gonorrhea, *(Neisseria gonorrhoeae)*, is indeed polyploid.

*N. gonorrhoeae* successfully infects at-risk individuals by reshuffling the genetic code for proteins expressed on its hairlike “pili” protrusions through a process called gene conversion. During gene conversion, one gene incorporates DNA from a variant copy that carries slightly different information. The high frequency of gene conversion events enhances pili protein diversity, which increases the likelihood that the human immune system won't detect the pathogen. It is conceivable that *N. gonorrhoeae* might briefly express two copies of the pilin gene in one cell after DNA replication, before cell division. But Tobiason and Seifert thought that the high frequency of gene conversion required for antigenic diversity might instead be supported by the presence of extra genome copies.

To explore this possibility, the researchers had to distinguish the amount of DNA associated with genome duplication prior to cell division from that found in a nondividing cell. They labeled the DNA of *N. gonorrhoeae* with fluorescent markers and, as a source of comparison, did the same for *E. coli*, which has provided a wellspring of knowledge on chromosomal DNA replication. Cells were either left untreated, allowing exponential growth, or were treated with an antibiotic that inhibits new rounds of replication but allows any DNA replication already in progress to finish. Cells in “stationary phase”—during which cell division stops and no new DNA replication begins, but active replication forks complete their task—were also labeled. Both antibiotic treated cells and stationary cells produce fully replicated chromosomes.

Using a standard cell sorting technique called flow cytometry, the researchers separated cells based on their DNA content. Treated and stationary phase *E. coli* cells produced individual cells with the equivalent of two, four, or eight genomes per cell. Since multiple initiation events on the chromosome can occur per round of cell division, the number of replicated genomes reflects the number of replication forks active when cell division stops. Untreated *E. coli* cells, however, revealed a wide range of DNA content per cell, likely reflecting the presence of multiple replication forks at various stages of replication and cell division. The DNA content of untreated gonococcal cells also varied, with most cells having between four and six genome equivalents.

Gonococcus exists as single (monococcal) and double spheres (diplococcal)—it is these two forms, Tobiason and Seifert show with fluorescence microscopy, that explains the range of DNA content in different cell populations found with flow cytometry. Monococci had, on average, about three to six genome equivalents, while diplococci averaged about four to ten per cell. To explore the reason for the expanded DNA content, the researchers treated gonococcal cells with antibiotics to generate fully replicated chromosomes. These experiments suggested that actively dividing cells have two or four pairs of active replication forks. They used independent measures to confirm that both monococci and diplococci contain fully replicated chromosomes with a single pair of active replication forks on each chromosome—which means that the DNA content they saw in gonococcal cells is not the result of multiple replication initiation events on a single chromosome, as occurs with *E. coli*, but represents multiple, fully replicated chromosomes. Future studies will have to determine whether polyploidy underlies pilin antigenic variation and the bacterium's success as a human pathogen. But this study leaves little doubt that it's time to retire the notion that all prokaryotes are haploid.

## 

**Figure:**
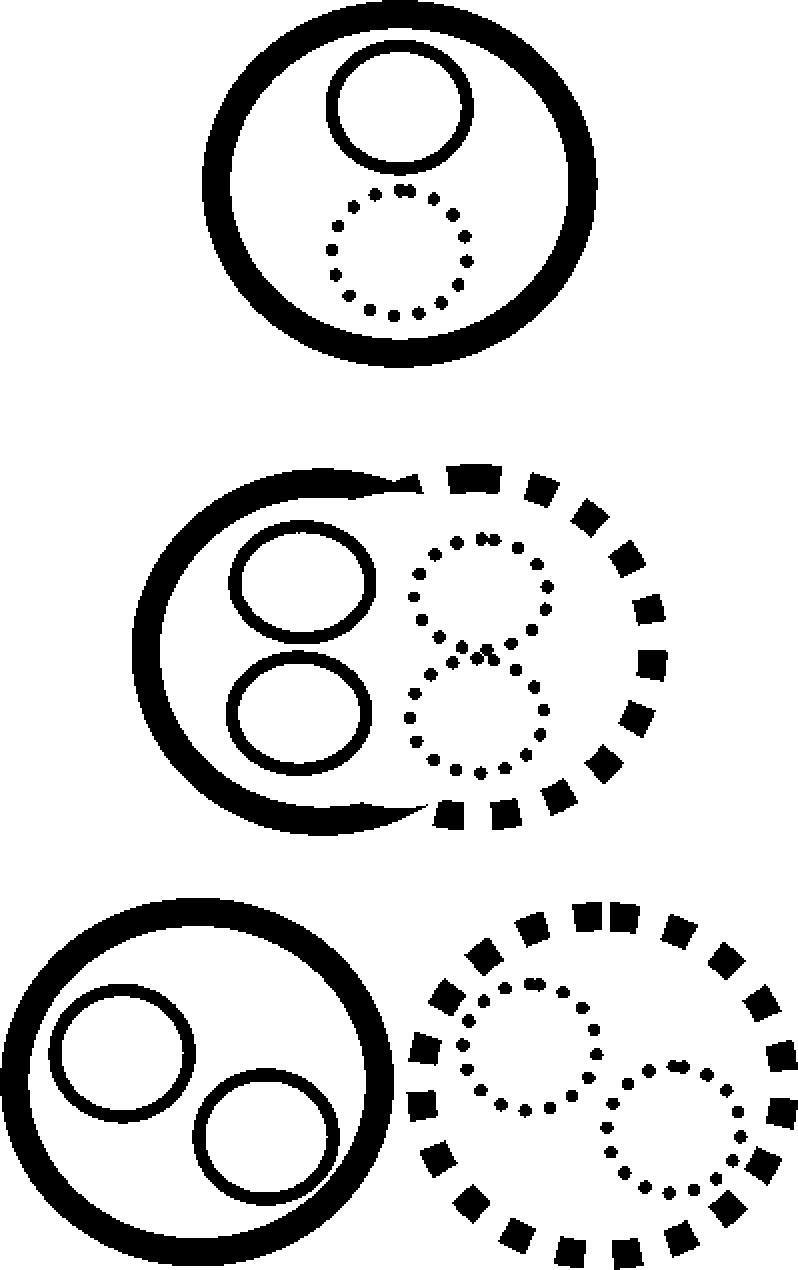
The obligate human pathogen, Neisseria gonorrhoeae, has multiple genome copies per cell, which may be necessary for antigenic diversity and survival within the human host.

